# Relations Between Dual Filial Piety and Life Satisfaction: The Mediating Roles of Individuating Autonomy and Relating Autonomy

**DOI:** 10.3389/fpsyg.2019.02549

**Published:** 2019-11-29

**Authors:** Peizhen Sun, Xiaoyue Fan, Yudi Sun, Hongyan Jiang, Lu Wang

**Affiliations:** ^1^Department of Psychology, School of Education Science, Jiangsu Normal University, Xuzhou, China; ^2^Department of Marketing, School of Management, China University of Mining and Technology, Xuzhou, China

**Keywords:** reciprocal filial piety, authoritarian filial piety, individuating autonomy, relating autonomy, life satisfaction

## Abstract

Filial piety (FP) was formerly a Confucian concept that specifies how children should treat their elders. In recent years, some psychologists have postulated that there are considerable overlaps between Chinese FP and notions found in other cultures. They have redefined FP as a contextualized personality emphasizing the psychological schema of parent-child interaction so that it fits universal cultural contexts. Based on this theory construction, this study aimed to examine the effects of reciprocal FP and authoritarian FP on life satisfaction and the mediating roles of individuating autonomy and relating autonomy therein. To do so, we recruited and surveyed 360 high school students in China. Subsequently, a mediation model based on the Dual Filial Piety Model and previous studies was tested. Results demonstrate that reciprocal FP predicted life satisfaction positively and that both individuating autonomy and relating autonomy played significant mediating roles in the relationship between reciprocal FP and life satisfaction. Moreover, authoritarian FP had a negative indirect effect on satisfaction through the mediating role of individuating autonomy, while authoritarian FP had a positive indirect influence on satisfaction through the mediating role of relating autonomy. The theoretical and practical implications of these findings are discussed.

## Introduction

Filial piety (FP) was formerly defined as a notion that specifies how children should treat their elders (Wong et al., 2010). It usually includes a variety of material and affective demands on children, such as love, respect, gratitude, obedience, support, and so on (Ho, 1996; Ho et al., 2012). In the early stages of Chinese history, FP emphasized the ethical principle of favoring the intimate, which described a parent-child interaction pattern motivated by natural affections between parents and children. From the time of the Han Dynasty (202 BC–220 AD), however, the emphasis of FP changed to the ethical principle of favoring the superior, which requires children to submit to hierarchical authority. This trend continued to the end of the Qing Dynasty (1911). In modern times, as political reform, marketization, and changes in people’s values have taken root, the emotionality in FP has re-acquired recognitions and obedience to authority is no longer the concept’s main focus (Bedford and Yeh, 2019). As global cultures continue to blend, the concept of FP has continued to shift. Bedford and Yeh (2019) redefined FP as a contextualized personality emphasizing the psychological schema of parent-child interaction, which has a universal function across cultures. FP is no longer a purely Chinese notion based on traditional Confucianism, but a psychological concept that focuses on intergenerational relations and applies to all cultural contexts.

In traditional Chinese society, FP has played a unique and positive role in the maintenance of family harmony and social stability. However, in the modern world, the effects of FP on the individual’s psychological development have been disputed by scholars. Some research has indicated that FP plays a positive role in the personal growth of children and parent-child relationships (Cheung et al., 1994), while others have indicated that FP suppresses self-expression and reduces children’s cognitive complexity (Ho, 1996). To resolve these inconsistent results, Yeh and Bedford (2003) proposed the Dual Filial Piety Model (DFPM). In this model, they differentiated two factors of FP: reciprocal FP and authoritarian FP. Reciprocal FP focuses on love, gratitude, care, and emotional intimacy expressed by children toward their parents, which resembles the general definition of “gratitude toward parents.” By contrast, authoritarian FP accentuates respect and obedience from children.

Previous studies have strongly indicated positive effects of reciprocal FP on individuals’ life satisfaction (e.g., Chen et al., 2016; Sun et al., 2016), but the influence of authoritarian FP on life satisfaction is still disputed (e.g., Leung et al., 2010; Yan and Chen, 2018). Therefore, this study examined the influences of both reciprocal FP and authoritarian FP on life satisfaction. There have been fewer studies conducted on the mechanisms underlying the relationship between FP and life satisfaction. Therefore, this study aimed to expand previous literature by investigating the roles of individuating autonomy and relating autonomy in the relationships between two kinds of FP and life satisfaction.

### Filial Piety and Life Satisfaction

Life satisfaction is an important indicator of well-being. It refers to the individual’s cognitive evaluation of the overall quality of his/her life (Diener, 1996). Previous research has yielded consistent results on the influence of reciprocal FP on life satisfaction. Several studies demonstrated that reciprocal FP exerts significantly positive effects on adolescent life satisfaction (e.g., Leung et al., 2010; Chen et al., 2016; Sun et al., 2016). For instance, Leung et al. (2010) showed that after statistically controlling for children’s ages, grade levels, and perceived parental warmth, reciprocal FP could predict higher teenager life satisfaction. Chen et al. (2016) showed that reciprocal FP is positively linked with an individual’s perceived quality of life.

While the relationship between FP and life satisfaction seems clear, the relationship between authoritarian FP and life satisfaction is still disputed. For instance, Leung et al. (2010) found that the regression coefficient from authoritarian FP to life satisfaction was negative when all other variables such as grade, age, and warmth were statistically controlled. Chen (2014) stated that the influence of authoritarian FP on life satisfaction was negative but non-significant. However, other studies have shown that the correlation coefficient between authoritarian FP and life satisfaction was positive (Sun et al., 2016; Yan and Chen, 2018). Yeh et al. (2013) demonstrated that the results on the effect of authoritarian FP on life satisfaction were inconsistent in different regions. We argue that although the coordination and family order implied in authoritarian FP may reduce parent-child conflict and promote family harmony, authoritarian FP may inhibit children’s self-expression, self-mastery, and individuality, which may have negative effects on life satisfaction. Thus, the overall impact of authoritarian FP on adolescents’ life satisfaction may be negative. Drawing on these arguments, we formed the following hypotheses:

***Hypothesis 1*:** Two sorts of FP will be significantly associated with life satisfaction.

***Hypothesis 1a*:** Reciprocal FP will be positively connected with life satisfaction.

***Hypothesis 1b*:** Authoritarian FP will be negatively connected with life satisfaction.

### The Roles of Individuating Autonomy/Relating Autonomy in the Relations Between Filial Piety and Life Satisfaction

Autonomy has been defined from two perspectives. From one angle, autonomy is defined as detachment and independence as opposed to reliance on others (Smetana et al., 2004). From another perspective, within the framework of Self-Determination Theory (SDT), autonomy means that an individual self-determines when he/she engages in actions and endorses the importance and value of those actions (Ryan and Deci, 2000). In that sense, people may act volitionally because the behaviors coincide with their authentic desires and internalized values (Ryan and Deci, 2000; Ryan et al., 2006). In line with SDT, the dual autonomy model (DAM) shares the notion that autonomy centers on obtaining self-identity. However, unlike SDT, DAM regards autonomy as an adaptive capacity that includes cognitive, functional, and emotional elements. It also differentiates two forms of autonomy: individuating autonomy and relating autonomy/inclusive autonomy (Gore and Cross, 2006; Yeh and Yang, 2006; Wu et al., 2015). Individuating autonomy is oriented toward acting volitionally by expressing individualistic attributes. In contrast, relating autonomy is oriented toward behaving volitionally with emphasis on the harmonious relationship between self and others.

Empirical studies have explored the link between two types of autonomy and FP (Hui et al., 2011; Yeh, 2014). For instance, Yeh (2014) stated that reciprocal FP predicted both individuating autonomy and relating autonomy positively, while authoritarian FP had a positive influence only on relating autonomy and a negative influence on individuating autonomy. It may be due to that the intimate affections between the youngsters and elders could provide a firm basis for autonomy development in children (Yeh, 2014). Children with high levels of reciprocal FP are likely to have a close relationship with their parents. Support and love from parents may promote children’s self-reflection and decision-making, which is conducive to the development of individuating autonomy and relating autonomy.

In contrast, authoritarian FP is concerned with obedience and hierarchical relationships (Yeh, 2003). Children with high levels of authoritarian FP may absorb parents’ opinions and suppress their own ideas, which may inhibit the development of individuating autonomy. Meanwhile, authoritarian FP requires children to respect and behave consistently with the beliefs of their parents. Children in strongly authoritarian FP households may interpret their parents’ views as guidance, and they may take parents’ opinions into account when they act or make decisions. They are likely to achieve individuals’ self-identity by behaving volitionally and emphasizing the harmonious relationships between themselves and their parents, thus demonstrating high levels of relating autonomy. Therefore, reciprocal FP will be positively associated with both individuating autonomy and relating autonomy, while authoritarian FP will be positively associated with relating autonomy but negatively associated with individuating autonomy.

Dual autonomy may also have a significant influence on life satisfaction. Previous studies proved that dual autonomy could play facilitating roles in an individual’s quality of life (e.g., Chirkov et al., 2003; Yeh and Yang, 2006; Rudy et al., 2007). Yeh and Yang (2006) stated that both individuating autonomy and relating autonomy were correlated with participants’ happiness. Similarly, Rudy et al. (2007) showed that both individual autonomy and inclusive autonomy were positively correlated to life satisfaction in different cultures, such as Chinese Canadian, European Canadian, and Singaporean culture.

Moreover, SDT and the DAM may provide evidences for the positive functions of dual autonomy. SDT maintains that there is a small set of universal psychological needs, including autonomy, competence, and relatedness (Deci and Ryan, 2008). If these psychological needs are consistently satisfied, people will experience well-being. Otherwise, they may experience unhealthy or dysfunctional psychological states (Ryan and Deci, 2000). According to SDT, autonomy as self-endorsement of actions could facilitate the satisfaction of the basic psychological needs which are considered innate “psychological nutriments” that all humans across cultures require to thrive (Sheldon et al., 2001).

Compared with SDT, the DAM elaborates on the positive relationship between dual autonomy and life satisfaction. The DAM holds that the essence of autonomy is the adaptive capacity to control situations and attain goals (Yeh and Yang, 2006; Wu et al., 2015). The realization of this ability will provide psychological and social adjustment (Yeh and Yang, 2006), which will facilitate life satisfaction. Specifically, individuating autonomy may elicit authentic expression of distinctiveness of self from others and allow for more complicated cognition and more flexibility in regulation (Yeh and Yang, 2006) such that the needs for autonomy and competence would be satisfied. By contrast, relating autonomy demands that children consider their parents’ views before making decisions, which may promote harmonious parent-child relationships (Yeh et al., 2007) such that the need for relatedness of children would be satisfied. All of this could help individuals with high levels of autonomy to maintain more life satisfaction.

In addition, the DFPM (Yeh and Bedford, 2003; Bedford and Yeh, 2019) may provide a theoretical explanation for the relationships between dual FP, dual autonomy, and life satisfaction. Yeh and Bedford (2003) proposed that two dynamic aspects of FP coexist: reciprocal FP and authoritarian FP. Reciprocal FP focuses on favoring the intimate, rooted in intimacy and genuine affections between parents and children, while authoritarian FP emphasizes respect for the superiors guided by obedience to role obligations. Bedford and Yeh (2019) further elaborated on the functions of two forms of FP from the perspective of parent-child interaction. They argued that two sorts of FP represent the horizontal--vertical duality of the parent-child relationship. More specifically, reciprocal FP is the horizontal dimension of the parent-child dyad and represents an affection-based interaction pattern between parents and children. In this pattern, children have a relationship of equality and positive communication with their parents, which could simultaneously meet mutual needs for relatedness and emotional safety of parents and children. Children with reciprocal FP beliefs are prone to have more positive interactions, more affective bonds, and more emotional safeties with their parents. Meanwhile, intimate affections and emotional safety could provide firm foundations for the development of individuating autonomy and relating autonomy (Yeh, 2014). Therefore, children with reciprocal FP beliefs are more likely to have high levels of individuating autonomy and relating autonomy, which in turn promote life satisfaction.

Authoritarian FP is the vertical dimension of the parent-child dyad, which represents an interaction pattern between parents and children based on family role hierarchy. In this pattern, children are asked to obey parents and put parents’ needs before their own. Children with more authoritarian FP beliefs are inclined to inhibit their own wills to comply with their parents’ ideas, which may suppress the development of individuating autonomy and therefore lower children’s life satisfaction. In addition, authoritarian FP requires that children respect and consider parents’ suggestions above their own ideas. Children with high levels of authoritarian FP are more likely to consider their parents’ opinions when making decisions, and thus may have high levels of relating autonomy, which increases their life satisfaction.

Based on the DFPM (Yeh and Bedford, 2003; Bedford and Yeh, 2019) and SDT (Deci and Ryan, 2008), and considering the evidence of the significant relationships between two sorts of FP and two types of autonomy (e.g., Hui et al., 2011; Yeh, 2014) and two types of autonomy and life satisfaction (e.g., Yeh and Yang, 2006; Rudy et al., 2007), we proposed the following hypotheses:

***Hypothesis 2*:** Two types of autonomy will be significant mediators in the association between two types of FP and life satisfaction.

***Hypothesis 2a*:** Both individuating autonomy and relating autonomy will play significant mediating roles in the relationship between reciprocal FP and life satisfaction.

***Hypothesis 2b*:** Both individuating autonomy and relating autonomy will have significant mediating roles in the connections between authoritarian FP and life satisfaction.

### The Current Study

In modern China, the connotation of FP is constantly changing with changes in society. The decline of obedience in the parent-child relationship in the present society redefines the norm of FP, especially authoritarian FP (Yan, 2016). In such a context, it is important to study the role of two sorts of FP in adolescent’s psychological development. Previous research has found the consistent results on the relationship between reciprocal FP and life satisfaction (e.g., Chen et al., 2016; Sun et al., 2016), but the effect of authoritarian FP is still controversial (e.g., Leung et al., 2010; Yan and Chen, 2018). Therefore, this study aimed to probe the effects of both reciprocal FP and authoritarian FP on adolescents’ life satisfaction.

Second, the mechanisms underlying the relationships between two types of FP and life satisfaction had been studied less. To the knowledge of the authors, no previous study had focused on the role of individuating autonomy and relating autonomy in the relationships between two types of FP and life satisfaction. Based on the DFPM (Yeh and Bedford, 2003; Bedford and Yeh, 2019), SDT (Ryan and Deci, 2000), and empirical research on the relationships among the above variables (Yeh and Yang, 2006; Rudy et al., 2007; Hui et al., 2011; Yeh, 2014), the current study proposed that individuating autonomy and relating autonomy might be the potential mediating mechanisms linking FP and life satisfaction. This study therefore intended to use SEM to examine these mediating effects.

Finally, the high school period is a significant stage for human development, during which students’ moral ideas are in a critical period of formation, promotion, and development. It is extremely important to examine FP and its role in the psychological development of high school students. Therefore, the present study selected high school students as our survey samples.

## Methods

### Participants and Procedures

We randomly recruited the participants from three high schools in a northern city of China. A total of near 400 questionnaires were distributed, and 90% (*n* = 360) returned the questionnaires. Overall, there were 193 male and 167 female participants, whose age ranged from 15 to 19 years (*M*_age_ = 17.21 years, SD = 1.67).

This study was approved by the Institutional Review Board at Jiangsu Normal University. Consistent with Institutional Review Board procedures, we first contacted the administrators in high schools and got the consents for their students’ participation in our study. Then, we obtained the informed consent from all participants (and their parents, in case the participants were under 16 years). After that, the participants filled in the questionnaires in the classrooms. All questionnaires were anonymous. It took about 20 min to complete all the tests.

### Measures

#### Dual Filial Piety

FP was measured *via* a 16-item Dual Filial Piety Scale (DFPS, Yeh and Bedford, 2003). The DFPS consists of two subscales: reciprocal FP (RFP; e.g., “be grateful to my parents for raising me”) and authoritarian FP (AFP; e.g., “give up one’s aspiration to meet the expectations of parents”). The DFPS is scored on a six-point Likert-type scale (1 = strongly disagree, 6 = strongly agree) with higher scores indicating stronger FP beliefs. The reliability in this study was acceptable for both the RFP subscale (Cronbach’s alpha = 0.73) and the AFP subscale (Cronbach’s alpha = 0.79).

#### Individuating Autonomy and Relating Autonomy

We used the Short Form of Adolescent Autonomy Scale (AAS-SF; 12 items) developed by Yeh (2014), whose items were extracted from the original version of AAS (Yeh et al., 2007), to measure two types of autonomy. The scale comprises of two six-item subscales measuring individuating autonomy (IA; six items) and relating autonomy (RA; six items). Items such as “I think it’s important for one to be independent and separate from parents” and “I’m always confident in my own decisions” were designed to assess IA. Items such as “I feel more confident about a decision when taking my parents’ suggestions into consideration” and “I try to coordinate with my parents to resolve things even when we disagree” were designed to assess RA. Participants responded on a five-point Likert-type scale from “strongly disagree” to “strongly agree”. A higher subscale score indicates a greater individuating autonomy or relating autonomy. The reliability of the scale in this study was good for both the IA subscale (Cronbach’s alpha = 0.75) and the RA subscale (Cronbach’s alpha = 0.89).

#### Life Satisfaction

Life satisfaction was assessed by Satisfaction with Life Scale (SWLS, Diener et al., 1985). In this study, we adopted the Chinese version of SWLS translated by Wu and Yao (2006), which has been widely used and proved to have a good reliability and validity in Chinese population (Wu and Yao, 2006; Yan and Chen, 2018). The SWLS is scored on a seven-point Likert-type scale (1 = strongly disagree and 7 = strongly agree). Participants were asked to rate their agreement with the statement of each item, for instance, “So far, I have gotten the important things I want in life.” The reliability of the SWLS was considered high in the current study (Cronbach’s alpha = 0.87).

### Data Analysis

We used the Structural Equation Modeling (SEM) to test the mediating effects in this study. The parceling method of “factorial algorithm” proposed by Rogers and Schmitt (2004), also known as “item-to-construct balance” (Little et al., 2002), was adopted to form the indicators of the latent variables in SEM. The parceling method is very popular and has been proved to be a valid method to reduce the observational error in SEM (Little et al., 2002).

The following indices were used to assess the model fit: *χ*^2^ fit index, Comparative Fit Index (CFI), Tucker-Lewis Fit Index (TLI), Root Mean Square Error of Approximation (RMSEA), and Standardized Root Mean Square Residual (SRMR). The fits are considered acceptable when the values of CFI and TLI are greater than 0.90, and values of RMSEA and SRMR are less than 0.08 (Marsh et al., 2004).

## Results

### Correlation Matrix Among Study Variables

Table 1 demonstrates the correlation coefficients and descriptive statistics for all the variables. Results showed that reciprocal FP was positively correlated with individuating autonomy, relating autonomy, and life satisfaction. In contrast, authoritarian FP was positively related to relating autonomy, but negatively correlated with individuating autonomy and life satisfaction. The results also supported the positive relationships among individuating autonomy, relating autonomy, and life satisfaction. In addition, the results indicated that gender had a significant effect on reciprocal FP (*t* = 3.05, *p* < 0.01, Cohen’s *d* = 0.36). Females scored higher than males on reciprocal FP (Female: mean = 43.75, SD = 4.16; Male: mean = 42.37, SD = 4.40). However, gender difference in authoritarian FP was not statistically significant (*t* = 0.37, *p* > 0.05, Cohen’s *d* = 0.04; Female: mean = 29.01, SD = 5.82; Male: mean = 28.80, SD = 5.17). These findings were consistent with previous research suggesting that females held stronger reciprocal FP than did Chinese males (Leung et al., 2010; Chen and Wu, 2017).

**Table 1:** Means, standard deviations, and correlation matrix for all variables.

	1	2	3	4	5
1. Reciprocal filial piety	1				
2. Authoritarian filial piety	0.11^*^	1			
3. Individuating autonomy	0.23^***^	−0.28^***^	1		
4. Relating autonomy	0.44^***^	0.33^***^	0.22^***^	1	
5. Life satisfaction	0.40^***^	−0.12^*^	0.29^***^	0.38^***^	1
Mean	43.01	28.90	24.49	26.89	23.54
SD	4.34	5.47	3.56	4.34	5.14

* p < 0.05;

*** *p < 0.001*.

### The Measurement Model (Confirmatory Factor Analysis)

First, a measurement model (Model 1) was developed using confirmatory factor analysis. In Model 1, there were five latent variables (reciprocal FP, authoritarian FP, individuating autonomy, relating autonomy, and life satisfaction) and 10 observable indicators. An initial test of Model 1 revealed a satisfactory fit to the data, with *χ*^2^ (25) = 57.38, *p* < 0.05; RMSEA = 0.060; SRMR = 0.041; TLI = 0.92; and CFI = 0.95. The loading coefficients from latent variables to their corresponding observable variables in Model 1 were all significant, indicating that the latent variables were all well represented by their corresponding indicators.

### The Structural Model

#### Testing the Total Effects of Two Sorts of Filial Piety on Life Satisfaction

We built a structural model (Model 2) that included the predictive variables (reciprocal FP, authoritarian FP) and the outcome variable (life satisfaction) without the mediating variables. This model fit well to the data, *χ^2^* (6) = 8.69; RMSEA = 0.035; SRMR = 0.038; TLI = 0.97; CFI = 0.97. The results indicated that the total effect of reciprocal FP on life satisfaction was significantly positive (*b* = 0.52, *p* < 0.001), but the total effect of authoritarian FP on life satisfaction was not significant (*b* = −0.09, *p >* 0.05). As the mediating effect of the mediating variable in the link between independent variable and dependent variable can still exist even if the total effect of independent variable on dependent variable is not significant (MacKinnon et al., 2000), in the following test, we will continue to examine the mediating effect of autonomy not only in the relationships between reciprocal FP and life satisfaction but also in the link between authoritarian FP and life satisfaction.

#### Testing the Mediating Effects of Individuating Autonomy and Relating Autonomy

**Figure 1:**
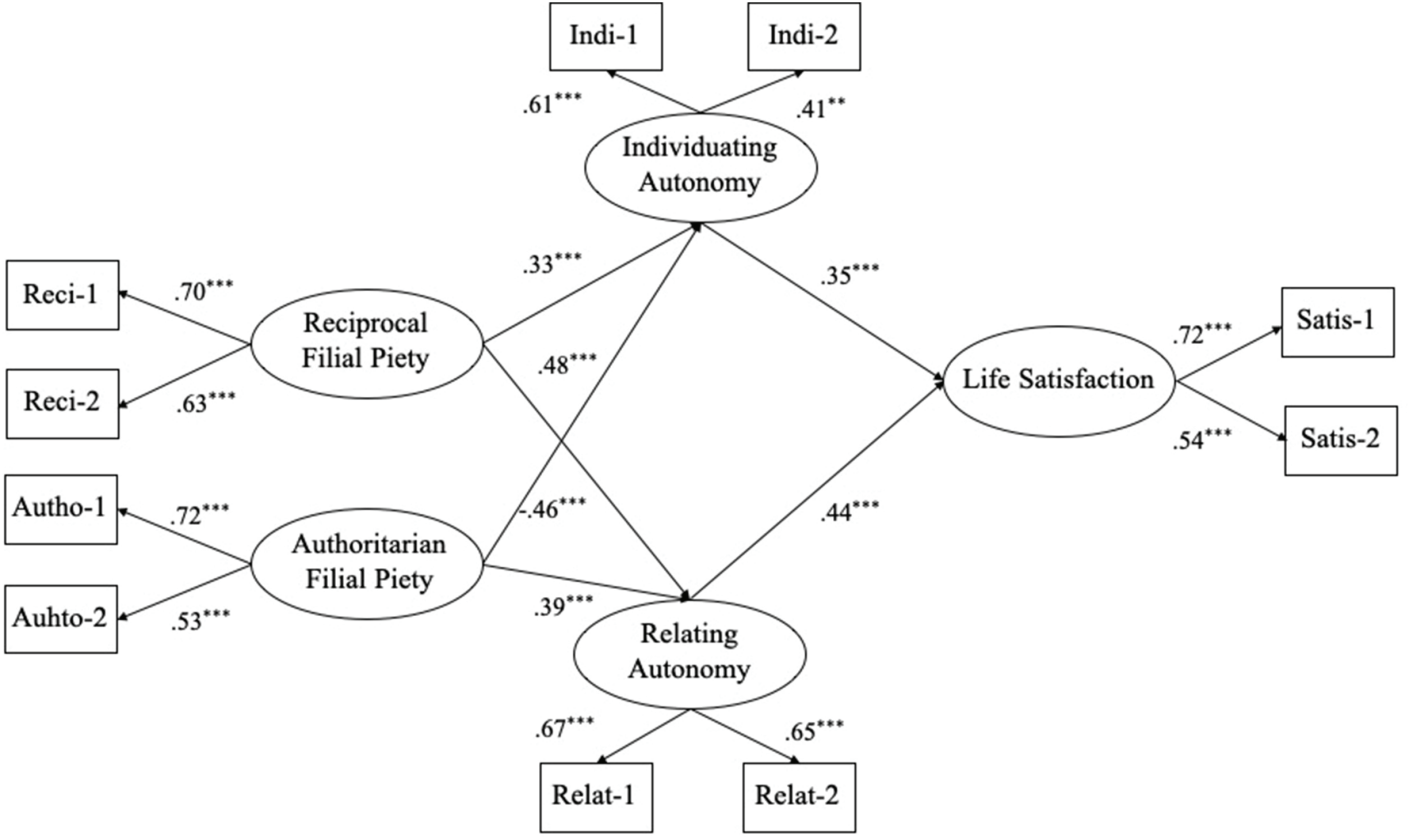
Final structure model with standardized estimates (Model 3). *Note*: Reci-1, Reci-2, Autho-1, Autho-2, Indi-1, Indi-2, Relat-1, Relat-2, Satis-1, and Satis-2 are indicators of their corresponding latent variables developed by using the method of item parceling.

To test the mediating effects of individuating autonomy and relating autonomy, we built and tested two alternative models: Model 3 (full-mediated model) in which two sorts of FP and life satisfaction were indirectly related through individuating autonomy and relating autonomy, and Model 4 (partially mediated model) with direct and indirect paths from two sorts of FP to life satisfaction. A test of Model 3 showed a good fit to the data, with *χ*^2^ (28) = 67.31, *p* < 0.05; RMSEA = 0.063; SRMR = 0.044; TLI = 0.91; CFI = 0.94. Meanwhile, most of the fit indices of the alternative Model 4 were acceptable except TLI, with *χ*^2^ (26) = 68.13, *p* < 0.05; RMSEA = 0.067; SRMR = 0.049; TLI = 0.88; CFI = 0.93. Further testing indicated that the Chi-square differences between Model 3 and Model 4 were not significant, with Δ*χ^2^* (2) = 0.82, *p* > 0.05. It can be seen that one fit index of Model 4 was not up to standard (TLI < 0.90). Moreover, Model 3 was simpler than Model 4, and Model 3 was more in line with the simplicity principle of structural equation model (Marsh et al., 2004). Therefore, we chose Model 3 as the final model (Figure 1).

Then, we adopted the bootstrapping procedure to examine the mediating effects in the final model (Model 3). The bootstrapping method involves repeated sampling of the dataset and estimating the indirect effect in each re-sampled dataset; the mediating effect is significant if the 95% confidence interval of the average estimates does not include zero (MacKinnon et al., 2004). The results indicated that the paths from reciprocal FP to both individuating autonomy and relating autonomy were all significantly positive (Figure 1). The paths from both individuating autonomy and relating autonomy to life satisfaction were also significantly positive. By contrast, authoritarian FP had a negative effect on individuating autonomy but a positive effect on relating autonomy (Figure 1). Furthermore, the bootstrapping method revealed that both individuating autonomy and relating autonomy played positive mediating roles in the relationships between reciprocal FP and life satisfaction. However, individuating autonomy played a negative mediating role while relating autonomy played a positive mediating role in the relationships between authoritarian FP and life satisfaction (see Table 2). The bootstrap results also showed that the differences between the mediating effects of relating autonomy and the mediating effects of individuating autonomy were significant (see Table 2), indicating that the mediating effects of relating autonomy were stronger than that of individuating autonomy.

**Table 2:** Indirect effects and 95% confidence intervals for model 3.

Model pathways	Estimated	95% CI	
		Lower	Upper
RFP → RA → LS	0.21	0.13	0.28
AFP → RA → LS	0.17	0.09	0.25
RFP → IA → LS	0.12	0.05	0.19
AFP → IA → LS	−0.16	−0.23	−0.08
*β*_**(RA-IA)**_ = (total indirect effect of RA) − (total indirect effect of IA)	0.61	0.13	1.11

*RFP, reciprocal filial piety; AFP, authoritarian filial piety; IA, individuating autonomy; RA, relating autonomy; LS, life satisfaction*.

We used the Method of Auxiliary Variable to examine *β*_**(RA-IA)**,_ in which we could only get the unstandardized coefficients. The estimated indirect effects in this table were all standardized coefficients except *β*_**(RA-IA).**_

## Discussion

The value of research on FP is being increasingly recognized (Chao and Tseng, 2002) and scholarship in this field is increasing. However, the roles of FP in the psychological development of individuals have not been fully explored. The present research sought to explore the effects of dual FP on adolescent life satisfaction and the underlying mediating roles of individuating autonomy and relating autonomy therein.

This study has certain theoretical implications. First, the current findings contribute to the literature by revealing the different effects of two types of FP on life satisfaction, which adds new evidence to the existing DFPM (Yeh and Bedford, 2003; Bedford and Yeh, 2019). The study also adds external validity by investigating the effects of FP on a different cultural context and age group (i.e., mainland Chinese adolescents). It should be noted that there is no consensus about the influences of dual FP on life satisfaction. Although scholars have found the consistent results on the effects of reciprocal FP on life satisfaction, the influence of authoritarian FP on life satisfaction is still ill-defined and controversial. This study shows that reciprocal FP has a positive influence on students’ life satisfaction, which is consistent with previous studies (Chen, 2014; Sun et al., 2016; Chen et al., 2018). Furthermore, this study demonstrates that the total effect of authoritarian FP on adolescent’s life satisfaction is non-significantly negative, which is consistent with Chen (2014). The reason for this may be that authoritarian FP itself is a complex concept with both functional and dysfunctional aspects that may complicate individuals’ adjustment development (Yeh and Bedford, 2003, 2004; Yeh, 2006; Yeh et al., 2013). To be more specific, authoritarian FP may generate family orders that reflect its functional aspects, but it may also suppress children’s personalities, individual autonomy, and self-expression, a reflection of its dysfunctional aspects (Schwartz et al., 2010). These two different effects may cancel each other out when they are considered together, leading to a non-significant overall effect on life satisfaction.

However, Yan and Chen (2018) indicated that authoritarian FP has a positive influence on life satisfaction, which is inconsistent with our results and the research of Chen (2014). Comparing our results with previous studies (Chen, 2014; Yan and Chen, 2018), we speculate that the relationship between authoritarian FP and life satisfaction may be different in different situations and among different populations (Yeh, 2006). Specifically, when highly authoritarian FP, which emphasizes respect for superiors and obedience to parents’ demands, is accompanied by intimate parent-child relationships, children may perceive parental authority as love and care. In this context, the effects of authoritarian FP may be functional and positive. On the contrary, when high authoritarian FP is accompanied by indifferent parent-child relationships, children may perceive parental authority as a restriction. In this context, the role of authoritarian FP may be dysfunctional and negative (Yeh, 2006).

Second, this study deepens scholarly understanding of the mediating mechanism that links FP to life satisfaction. To the best knowledge of the authors, this is the first study that has tested the mediating roles of individuating autonomy and relating autonomy in the relationships between dual FP and life satisfaction. We found that dual autonomy (individuating autonomy and relating autonomy) played significant roles in the relationship between two types of FP and life satisfaction. This implies that the effects of reciprocal FP and authoritarian FP on life satisfaction can be mediated by the roles of individuating autonomy and relating autonomy. On one hand, reciprocal FP facilitates two types of autonomous development, which is consistent with the latest research of Yeh (2014), but is not completely consistent with the earlier findings of Yeh et al. (2007). Yeh et al. (2007) used a structural equation model in which individuating autonomy was an independent variable and reciprocal FP was the dependent variable. This study finds that individuating autonomy had a negative effect on reciprocal FP. This indicated that individuals with stronger individuating autonomy tend to express individualistic attributes that may contradict Chinese parents’ collective belief and easily trigger parent-child conflict, thus inhibiting reciprocal FP (Yeh et al., 2007). By contrast, Yeh (2014) adopted a new perspective to build another model in which reciprocal FP was an independent variable and individuating autonomy was the dependent variable. This time, reciprocal FP was found to favor individuating autonomy. In our study, we built the model according to Yeh (2014), and our results were consistent with those of Yeh (2014). This indicates that individuals with high reciprocal FP may experience more parental care and therefore be more inclined to reflect profoundly and decide independently, which promotes individuating autonomy (Yeh, 2014). The results indicated that intimate affection from reciprocal FP could indeed offer a firm basis for the development of autonomy (Yeh, 2014), which in turn significantly affects life satisfaction.

On the other hand, authoritarian FP has different functions on two types of autonomy. It can promote relating autonomy but also inhibit individuating autonomy, which is consistent with the findings of Yeh (2014). As mentioned above, authoritarian FP is a mixed variable with two connotations: positive family order and negative blind obedience (Yeh, 2003). Authoritarian FP emphasizes respect and compliance from children toward their parents (Yeh and Bedford, 2003). The principle of authoritarian FP requires children to consider their parents’ opinions when making decisions (Liu, 2013), which facilitates the development of relating autonomy. Simultaneously, authoritarian FP may suppress children’s thoughts and personality (Chen, 2014), which may undermine children’s individuating autonomy, and, in turn, lower children’s life satisfaction.

This study also shows that the mediating effect of relating autonomy was stronger than that of individuating autonomy. According to the DAM (Yeh and Yang, 2006), autonomy is an adaptive capacity that can be enacted through two orientations: individuating autonomy and relating autonomy. Individuating autonomy and relating autonomy can coexist within an individual and may co-promote individuals’ psychological adaptation and well-being. DAM further proposed the Domain Superiority Hypothesis, which states that individuating autonomy is more strongly associated with intrapersonal domain variables, while relating autonomy is more associated with interpersonal domain variables (Yeh and Yang, 2006; Yeh et al., 2009; Wu et al., 2015). FP represents a parent-child interaction pattern between parents and children (Bedford and Yeh, 2019), and thus belongs to interpersonal domain variable. Therefore, compared with individuating autonomy, relating autonomy has stronger connections with FP and plays a stronger mediating role in the relationship between two types of FP and life satisfaction. In addition, China is a collectivist culture in which relationship harmony is given greater importance (Kwan et al., 1997). Chinese adolescents may value harmonious interpersonal relationships with others more than adolescents of other cultures. Adolescents are more inclined to consider their parents’ views before making decisions, and relating autonomy may play a more important role in adolescents’ psychology and social adaption. Thus, it is reasonable to postulate that the mediating effect of relating autonomy is stronger than that of individuating autonomy among Chinese adolescents.

Although our study contributes new insight into the empirical literature by confirming the aforementioned results, it also has some limitations. First, our sample students were mainly from high schools in China, so the generalizability of our results to other demographic groups may be limited. Future investigations should examine topics such as FP, autonomy, and well-being in other cultural contexts to explore potential differences across cultures. Second, this study had a cross-sectional design that limited causal inferences. Researchers in the future may choose to adopt experimental or longitudinal designs to probe the possible cause-and-effect relationships between FP and well-being. Finally, this study focused on the mediating role of only dual autonomy in the relationships between two types of FP and life satisfaction without investigating the moderation effects therein. Our study therefore did not completely resolve the debate about the effect of authoritarian FP on life satisfaction. Testing the roles of certain potential moderating variables, such as parent-child relationship and parent-child attachment, in the relationship between authoritarian FP and life satisfaction in future studies may provide further insights.

## Data Availability Statement

The datasets generated for this study are available on request to the corresponding author.

## Ethics Statement

The studies involving human participants were reviewed and approved by the Institutional Review Board of the Jiangsu Normal University. Written informed consent to participate in this study was provided by the participants’ legal guardian/next of kin.

## Author Contributions

PS designed, wrote, and approved all contributions to the study. XF participated in designing the study. YS participated in reviewing the literature and carried out all the analyses for the study. LW participated in collecting the data. HJ helped to design the study and edit the manuscript.

### Conflict of Interest

The authors declare that the research was conducted in the absence of any commercial or financial relationships that could be construed as a potential conflict of interest.
